# Extrinsic Calibration of Multiple Two-Dimensional Laser Rangefinders Based on a Trihedron

**DOI:** 10.3390/s20071837

**Published:** 2020-03-26

**Authors:** Fei Zhu, Yuchun Huang, Zizhu Tian, Yaowei Ma

**Affiliations:** School of Remote Sensing and Information Engineering, Wuhan University, Wuhan 430072, China

**Keywords:** 2D laser rangefinder, extrinsic calibration, trihedron, weighted iterative, Perspective-Three-Point (P3P)

## Abstract

Multiple two-dimensional laser rangefinders (LRFs) are applied in many applications like mobile robotics, autonomous vehicles, and three-dimensional reconstruction. The extrinsic calibration between LRFs is the first step to perform data fusion and practical application. In this paper, we proposed a simple method to calibrate LRFs based on a corner composed of three mutually perpendicular planes. In contrast to other methods that require a special pattern or assistance from other sensors, the trihedron corner needed in this method is common in daily environments. In practice, we can adjust the position of the LRFs to observe the corner until the laser scanning plane intersects with three planes of the corner. Then, we formed a Perspective-Three-Point problem to solve the position and orientation of each LRF at the common corner coordinate system. The method was validated with synthetic and real experiments, showing better performance than existing methods.

## 1. Introduction

Lasers can accurately obtain the depth information of the surrounding environment and form a high-precision point cloud. Thus, these devices have been widely applied to various tasks, such as location [1], navigation [2], three-dimension reconstruction [3], etc. Among them, 3D laser scanners (LSs) and 2D laser rangefinders (LRFs) are the most widely used devices. LSs can obtain the dense 3D point clouds of the surrounding by shooting and receiving laser pulses in three-dimensional space. Similarly, a combination of multiple LRFs generates laser points in different directions simultaneously, which can be fused into 3D cloud points. The most important advantages are that LRFs have the characteristics of smaller size, lower cost, and better stability than a 3D laser scanner [4]. Therefore, two or more LRFs are usually assembled on self-driving vehicles [5], industrial robots [6], and simultaneous localization and mapping (SLAM) devices as an alternative device of LSs [7]. To convert the LRFs’ raw data, which record the distance information in LRF coordinates into a unified world coordinate and form a dense 3D point cloud, the key step is to determine the relationship between different LRFs’ coordinates. This process is also called the extrinsic calibration between two LRFs.

Unlike an LS, which can obtain a dense 3D point cloud, multiple LRFs separately obtain the distance information in a 2D plane. Moreover, the spectrum of LRFs lies outside the visible light spectrum, resulting in the invisibility of LRF points. Therefore, it is difficult to find the corresponding points in three-dimensional space and directly determine the extrinsic parameters between the two coordinate systems of LRFs. In recent years, many scholars have proposed different methods to calibrate the extrinsic parameters between two or more LRFs. These methods can be divided into two groups according to whether a third-party sensor is used.

The first one is taking other sensors (such as camera, The Global Positioning System (GPS), odometer, etc.) as a bridge and finding the extrinsic parameters between multiple LRFs and this sensor. For example, Zhang [8] introduced the idea of calibrating the extrinsic parameters between a camera and an LRF by using a checkerboard as a pattern. Based on this method, Xie et al. [9] experimented with an autonomous vehicle that contained three LRFs and a camera. They calibrated the extrinsic parameters between three LRFs and a camera, and then calculated the extrinsic parameters between the three LRFs by using the mutual camera as a bridge. Bok et al. [10] proposed a different LRF-camera calibration method based on point-on-line constraints. Then, using the camera as an intermediary, he calibrated the extrinsic parameters between two LRFs without overlapping regions. Blanco et al. [11] applied the odometer data and multiple LRFs data to construct the multiple 3D point cloud of the same scene, and then completed the calibration between LRFs by 3D point cloud matching. Glas [12] and Schenk [13] et al. estimated the relative positions between LRFs by matching the trajectories of dynamic objects in the scene. Although these methods do not require any specially designed markers, the calibration error between the LRFs and the intermediate sensors may be accumulated in the calculation of the extrinsic parameters between the LRFs when they do not have enough common view.

Another method is to directly calibrate multiple LRFs with some specific markers in 2D point clouds. For instance, when multiple LRFs observe a V-shaped orthogonal plane, an obvious V-shaped polyline would be detected in the 2D point clouds formed by each LRF. According to the geometric relationship, the straight lines on the same surface of the V-shaped plane satisfy the coplanar equation, and two normal vectors of the V-shaped plane are perpendicular to each other, which are, respectively, called coplanar constraints and orthogonal constraints. Based on this, Fernandez-Moral [14] proposed a calibration method by observing an V-shaped orthogonal corner and forming V-shaped polylines in different LRFs’ coordinate systems. Adding all the equations of constraints as an optimization function, they optimized the extrinsic parameters between the LRFs by Levenberg–Marquardt (LM) algorithm [15]. However, this method needs an initial value and a large number of observations to get an accuracy calibration result. Yin et al. [16] further extended this method and replaced the V-shaped plane with a corridor scene. The benefit of this method was the reduced number of observations, but an initial value of the extrinsic parameters is still needed. Unlike these methods proposed in [14,16], Choi [17] proposed an algebraic method to solve the initial value. He first transformed all the coplanar constraints and orthogonal constraints obtained in different directions and positions into the forms of linear equations, then solved the initial value of the extrinsic parameters by the least-squares solution. Finally, he optimized the initial value by the LM algorithm. Although an initial value was avoided, the linear equations might degenerate in some circumstances. A sphere can also be used as a calibration pattern. When observing a sphere pattern with multiple LRFs, arcs can be detected in the 2D point clouds. By fitting the circle equation of the arcs, the center point of the sphere can be calculated in each LRFs’ coordinate systems. Then, the extrinsic parameters between the LRFs’ coordinate systems can be obtained. Pereira et al. [18] were the first to use a sphere to calibrate the extrinsic parameters between multiple 3D laser scanners. Furthermore, Chen et al. [19] applied it to the calibration of multiple LRFs. Although they claimed that the process of the calibration was fully automatic, they had to keep moving the position of the sphere to get enough matching points, which was very tedious. There were also some researches using special patterns. For example, Antone et al. [20] designed a special pattern like a pyramid which can determine the position of LRFs. Almeida et al. [21] used a cone to establish the relationship between an LS with an LRF. In recent years, more and more researchers have studied simple and flexible methods that can be applied in daily environment rather than in laboratory. For example, He et al. [22] calibrated multi-LiDAR system in an underground parking lot. However, this method needs the localization data of the platform provided by other sensors. Fernández et al. [23] proposed a method to calibrate a set of LRFs by observing a common planar surface from different orientations. Song et al. [24] calibrated two dual 2D laser rangefinders by a internal corner in the room, but it can only be used in the situation where two LRFs are set at the same plane. Sharifzadeh et al. [25] determined the accurate position of the LRFs relative to the robot using a single-plane as calibration target. The main problems of the above methods are that either a large number of observed data are needed, or an initial value of extrinsic parameters is needed.

The LRFs emit a laser pulse to the target, receive the echo pulse by the receiving system, and then calculate the target distance through the round-trip time [26]. The ranging noise of an LRF is mainly composed of the fixed deviation of the LRF and the random noise in the measurement process [27]. Among them, the fixed deviation of the LRF is a fixed value, and its influence can be removed by the previous LRF’s intrinsic calibration. Therefore, in this paper, we assumed that all the intrinsic parameters of the LRFs were well-calibrated and ignored the influence of the fixed deviation. The random noise in the measurement process is affected by the system response time, laser pulse bandwidth and external environment. To reduce the influence of random ranging noise, we assumed that the ranging noise was a random variable following Gaussian distribution [28]. In this assumption, we analyzed its error model and derived the mathematical form of a weighted iterative (WI) method of fitting a straight line with laser points. Then, we proposed a new flexible method taking a trihedral object as the calibration pattern, which is common in both indoor and outdoor scenes. The calibration steps were as follows, first, the world coordinates were established by taking the intersection point of trihedral as the origin and the three edges as the *X*, *Y*, and *Z* axes. Then, the position of the LRFs were adjusted so that the laser scanning plane intersects three mutually perpendicular planes of the trihedron to obtain discrete laser points. By fitting laser points on three planes into three intersecting lines, we got the parameters of the three intersecting lines. The three intersecting lines and the trihedral origin were used to construct a triangular pyramid, then we calculated the coordinates of the three intersecting points in the laser coordinate system and the world coordinate system. After that, a typical Perspective-Three-Point problem (P3P) [29] was introduced to determine the extrinsic parameters between the LRFs and the world coordinate system. Finally, the extrinsic parameters between LRFs were obtained through coordinate system transformation. The whole calibration process does not need the help of other sensors, a rough initial value, or plenty of observation data. The only thing we should do is set all the LRFs to observe a trihedron at the same time. Compared with previous calibration methods, the method proposed in this paper has the following three advantages.

1. First, compared with other methods, this method is more flexible, and it is very easy to find a trihedron satisfying the conditions in both indoor and outdoor scenes. The calibration between LRFs can be done quickly without the help of any other special manual calibration patterns or other sensors. The method can frequently check the calibration during the surveying process of mobile system.
2. Second, the method does not require multiple observations or initial values of extrinsic parameters, which simplifies the whole calibration process. As long as an LRF’s scanning plane intersects with three mutually perpendicular planes of the trihedron, it provides enough information to solve the extrinsic parameters between the LRF’s coordinate with the trihedron. Because the proposed method is simple, it can also act as the initial estimation for optimization.
3. Third, the proposed method can achieve better accuracy than previous methods. To improve the accuracy of the algorithm, the algorithm proposed in this paper considered the ranging noise of the LRFs and established the ranging noise model. By giving different weights to different points according to the ranging noise model, we deduced the weighted linear fitting equation. This trick ensured the accuracy of lines and improved the overall calibration accuracy. It also ensured the robustness of our method under different noise levels.

The rest of this paper is organized as follows. In Section 2.1, Section 2.2 and Section 2.3, we introduce the theoretical basis of our method including the trihedral model, the WI method, and the P3P problem. In Section 2.4, we introduce the basic processes and operations of calibrating extrinsic parameters between multiple LRFs. In Section 3.1, we carried out a great deal of simulation experiments comparing our method with traditional line-fitting methods to verify the accuracy and stability of our method. In Section 3.2, we observed a real trihedron with a SLAM device integrated with three LRFs to calibrate the extrinsic parameters between these LRFs and verify the feasibility of this method in the actual environment. Finally, in Section 4, the main work of this paper and the future work are summarized.

## 2. Calibration Approach

Our method can solve the extrinsic parameters between two LRFs with only one observation of a trihedron at the same time. The calibration between multiple LRFs can be thought as multiple repetitions of the calibration of two LRFs. Therefore, we take the calibration of two LRFs as an example to describe the calibrating processes.

**Figure 1:**
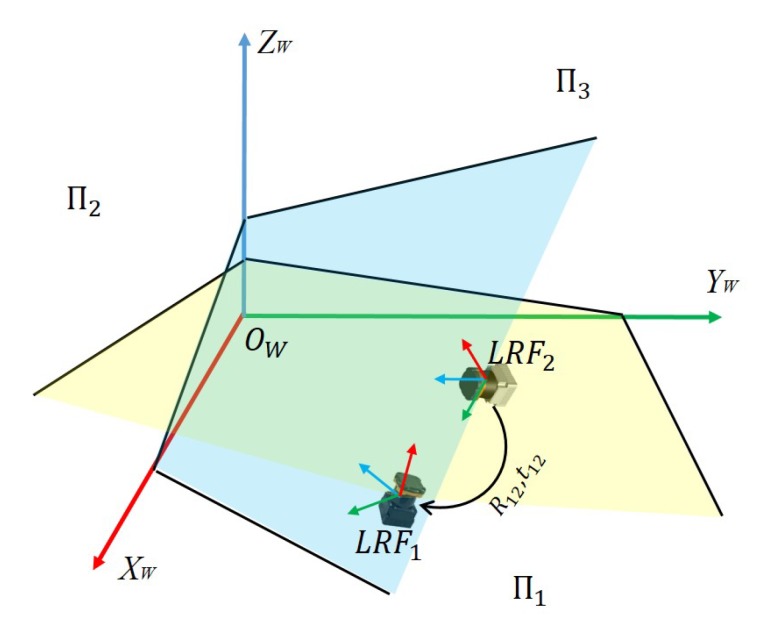
Calibration schematic diagram of two laser rangefinders.

As shown in Figure 1, three mutually perpendicular planes, $\Pi_{1}$, $\Pi_{2}$, and $\Pi_{3}$, constitute a trihedron. Setting three edges of the trihedron as *X*, *Y*, and *Z* axis and the vertex of the trihedron as the origin, we get the world coordinate system $O_{W}-X_{W}Y_{W}Z_{W}$. The two LRFs are, respectively, denoted as LRF1 and LRF2. We set the ranging center as the origin, and the scanning plane as x−o−y plane, as shown in blue and yellow plane in Figure 1. We represent the laser coordinate system of the LRFs as $O_{1}-X_{1}Y_{1}Z_{1}$ and $O_{2}-X_{2}Y_{2}Z_{2}$. Its extrinsic parameters to the world coordinate system are denoted as $\left[R_{W1}|t_{W1}\right]$ and $\left[R_{W2}|t_{W2}\right]$. A point is denoted as $P_{1}$, $P_{2}$, and $P_{W}$ in the two-laser coordinate systems and the world coordinate system, respectively; therefore, the extrinsic parameters between them can be expressed as

(1) $$\begin{array}{c} P_{W}=R_{W1}P_{1}+t_{W1}\\ P_{W}=R_{W2}P_{2}+t_{W2} \end{array}$$

Taking the world coordinate system as a bridge, we can derive the extrinsic parameters between the two LRFs coordinate systems. That is,

(2) $$T_{12}:\left\{\begin{array}{c} R_{12}={R_{W2}}^{-1}R_{W1}\\ t_{12}={R_{W2}}^{-1}\left(t_{W1}-t_{W2}\right) \end{array}\right.$$

where $R_{12}$, $t_{12}$, and $T_{12}$ represent the rotation matrix, translation vector, and transfer matrix between the LRF1 coordinate and LRF2 coordinate, respectively.

### 2.1. Trihedron Model

**Figure 2:**
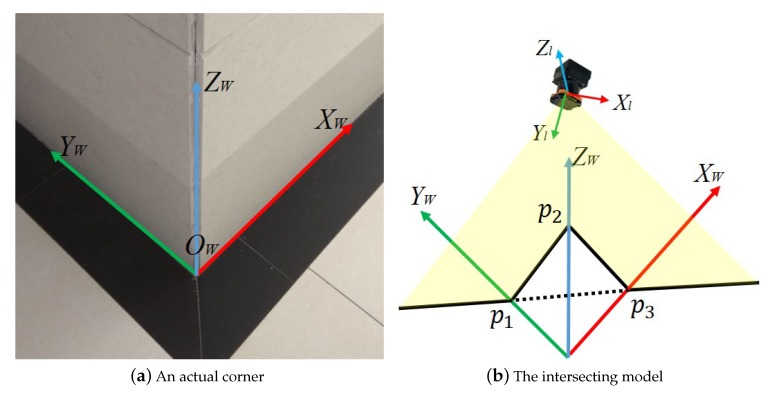
Trihedron model: (**a**) an actual corner and (**b**) the intersecting model of the LRF scanning plane with the corner.

The key step in calibrating the extrinsic parameters between two LRFs is determining the relationship between the two LRFs’ coordinate systems and the world coordinate system. Taking a single LRF as an example, the progress of determining the extrinsic parameters between the LRF coordinate system and the world coordinate system is divided into three main steps. As shown in Figure 2a, we set the world coordinate system based on a trihedron which is common in outdoor and indoor scenes. Figure 2b is an abstract illustration showing that an LRF is observing a trihedron. The LRF coordinate system and the world coordinate system are set as the description in Section 2. From Figure 2b, an LRF’s origin is in the third octants of the world coordinate system and the laser ranger scanning plane intebrsects with the three planes of the trihedron.

The basic principle of calibrating the extrinsic parameters between a single LRF and the trihedron is to find the coordinates of corresponding points in the laser coordinate system and the world coordinate system and then calculate the extrinsic parameters. As the trihedron model showed in Figure 2b, the scanning plane of the LRF intersects with the three planes of the trihedron; therefore, forming three intersecting lines, and three straight lines intersect with each other, which forms three intersecting points: $P_{1}$, $P_{2}$, and $P_{3}$. The coordinates of these points can be obtained in the laser coordinate system by fitting the laser points into lines. At the same time, the three points and the origin of the trihedron form a vertical pyramid in the world coordinate system, which can be regarded as a special form of P3P problem. By solving the P3P problem, the coordinates of the three points in the world coordinate system can be represented as $Q_{1}$, $Q_{2}$, and $Q_{3}$. Finally, the extrinsic parameters between the laser coordinate system and the world coordinate system are solved by the three corresponding points. The solution involves two main processes: the first step is fitting the laser points in the laser coordinate system and calculating the coordinates of the three intersecting points under the circumstance that there are ranging noise in laser points data. The second step is constructing and solving the P3P problem. Section 2.2 below describes the method of fitting a straight line considering the ranging noise in laser points data, and Section 2.3 describes the solution of this special P3P problem.

### 2.2. Weighted Iterative Method

The first step of calibrating the extrinsic parameters is to fit the laser points into three straight lines. Because of the scanning resolution and the ranging noise of the LRF, the laser points on the three planes of the trihedron are a collection of discrete distribution points, and these points are not strictly on a straight line. Fitting three lines requires determining the best equations of lines that are close to these discrete points. There are many different methods fitting laser points into lines, including classical Least Squares (LS) [30] method and Total Least Squares (TLS) [31] method. However, these methods do not consider the ranging noise of two-dimensional LRF points, resulting in low fitting accuracy line parameters. To reduce the effect of the ranging noise in fitting lines, we assume that the ranging noise of an LRF is a random variable following Gaussian distribution. Under this assumption, this paper proposed a weighted iterative method to fit the noised laser points into a line. The mathematical derivation of the weighted iterative method is shown as follows.

**Figure 3:**
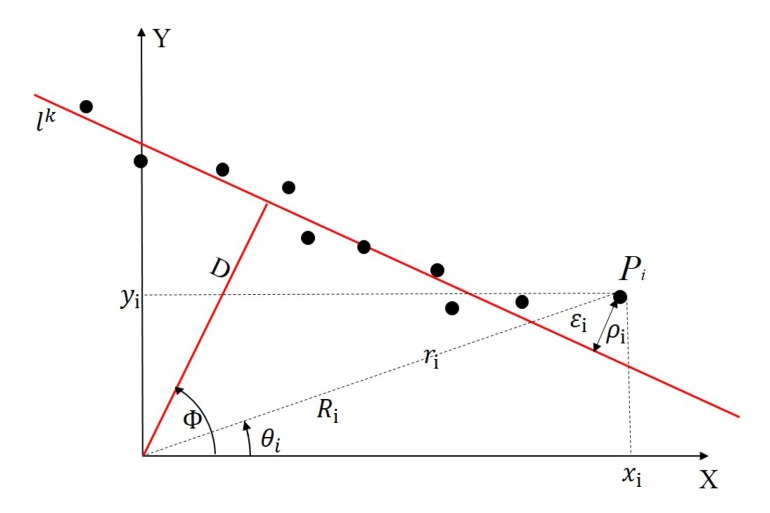
Schematic diagram of weighted iterative. A red line $l^{k}$ represents the intersection between the laser scanning plane and a plane of the trihedron. Some black points represent the laser points that do not coincide with the red line because of the influence of the ranging noise. The Cartesian coordinates system represents the scanning plane of the LRF.

As shown in Figure 3, a red line $l^{k}$ represents the intersecting line between the laser scanning plane and a plane of the trihedron. The black points represent the laser points that do not coincide with the red line because of the influence of the ranging noise. The Cartesian coordinates system represents the scanning plane of the LRF. Supposing the measuring distance between a laser point $P_{i}$ and the center of the LRF is $r_{i}$, because the distance contains the ranging noise of the LRF, it is the sum of the true distance $R_{i}$ and the random ranging noise $\varepsilon_{i}$. Among them, $\varepsilon_{i}$ is a random variable satisfying the Gaussian distribution with a mean of 0 and a variance of $\sigma_{r}^{2}$ [32].

(3) $$r_{i}=R_{i}+\varepsilon_{i}$$

Then, because the angle between the laser point and the *x* axis of the laser coordinate system is $\theta_{i}$, the Cartesian coordinates of the laser points can be expressed as $\left(x_{i},y_{i}\right)$.

(4) $$\left[\begin{array}{c} x_{i}\\ y_{i} \end{array}\right]=r_{i}\left[\begin{array}{c} \cos\theta_{i}\\ \sin\theta_{i} \end{array}\right]$$

Supposing the parameters of line $l^{k}$ as (D,Φ), which is unknown. Thus, the distance between the laser point $P_{i}$ and the line $l_{k}$ can be represented as $\rho_{i}$, that is,

(5) $$\rho_{i}=r_{i}\cos(\Phi -\theta_{i})-D$$

Substitute Equation (3) into Equation (5), then it can be transformed as

(6) $$\begin{array}{cc} \rho_{i} & =\left(R_{i}+\varepsilon_{i}\right)\cos\left(\Phi -\theta_{i}\right)-D\\ & =\varepsilon_{r}\cos\left(\Phi -\theta_{i}\right) \end{array}$$

If the ranging noises of the laser points are not considered, it means that the distance between each laser point to the line has the same weight. Thus, the cost function of fitting lines can be expressed as minimizing the sum of distances between a series of laser points and the line $l^{k}$.

(7) $$L\left(D,\Phi \right)=\arg\min_{D,\Phi }\sum_{i=1}^{N}\rho_{i}^{2}$$

Solving Equation (7) is a total least square problem and its solution has already been deduced in the literature [33]. Therefore, the result is given directly in Equations (8) and (9) without any derivation. To be distinct with real value, it is denoted as $\hat{D}$ and $\hat{\Phi }$.

(8) $$\hat{\Phi }=\frac{1}{2}\arctan2(-2\sigma_{xy},\sigma_{y}^{2},-\sigma_{x}^{2})$$

(9) $$\hat{D}=\bar{x}\cos\hat{\Phi }+\bar{y}\sin\hat{\Phi }$$

The function arctan2() represents the angle values of the arctangent function in the fourth quadrant and represents the mean values of the laser points in Cartesian coordinates system, and $\sigma_{xy}$, $\sigma_{x}^{2}$, and $\sigma_{y}^{2}$ represent the covariance and variance of the laser points. Their mathematical formulae are as follows.

(10) $$\sigma_{x}^{2}=\frac{1}{N}\sum_{i=1}^{N}{\left(x_{i}-\bar{x}\right)}^{2}$$

(11) $$\sigma_{y}^{2}=\frac{1}{N}\sum_{i=1}^{N}{\left(y_{i}-\bar{y}\right)}^{2}$$

(12) $$\sigma_{xy}=\frac{1}{N}\sum_{i=1}^{N}\left(x_{i}-\bar{x}\right)\left(y_{i}-\bar{y}\right)$$

(13) $$\bar{x}=\frac{1}{N}\sum_{i=1}^{N}x_{i}$$

(14) $$\bar{y}=\frac{1}{N}\sum_{i=1}^{N}y_{i}$$

Equations (8) and (9) are the parameters of a line under the condition that all laser points have equal weights. Actually, the distance measurement of each laser point is affected by random noises. In some applications, the effect of the random ranging noise can be ignored, but to accurately calibrate the extrinsic parameters between the two LRFs, it is necessary to take the random ranging noises of LRFs into account when fitting the straight lines. For each laser points, according to Equation (6), the variance of $\rho_{i}$ can be expressed as

(15) $$\sigma_{\rho_{i}}^{2}=E\left\{\rho_{i}\rho_{i}^{T}\right\}=\sigma_{r}^{2}\cos^{2}\left(\Phi -\theta i\right)$$

Equation (15) indicates that the variance of the distance between the laser points and the actual line is related to the LRF’s ranging variance, which can be obtained through intrinsic parameters calibration, the scanning angle of the laser points, and the angle parameter of a line. Therefore, to reduce the deviation of fitting a straight line with uncertain laser points, we took the reciprocal of $\sigma_{\rho i}^{2}$ as the weight of each laser points, and added it to the cost function Equation (7). The cost function of fitting a straight line is finally expressed as

(16) $$\begin{array}{cc} L(D,\Phi ) & =\arg\min_{D,\Phi }\sum_{i=1}^{N}\frac{\rho_{i}^{2}}{\sigma_{\rho i}^{2}}\\ \; & =\arg\min_{D,\Phi }\sum_{i=1}^{N}\frac{{\left(r_{i}\cos(\Phi -\theta_{i})-D\right)}^{2}}{\sigma_{r}^{2}\cos^{2}\Phi -\theta_{i}} \end{array}$$

Equation (16) is a nonlinear function related to *D* and Φ. To solve this function, the Coordinate ascent method was introduced to obtain the solution of this function [34]. First, substitute the initial value $\hat{\Phi }$ into the Equation (16), and the cost function becomes a convex function. Let $\frac{\partial L(D,\hat{\Phi })}{\partial D}=0$, we can get the solution of *D*

(17) $$D={\left(\sum_{i=1}^{N}\right)\frac{1}{\sigma_{\rho i}^{2}}}^{-1}\sum_{i=1}^{N}\frac{r_{i}\cos\left(\Phi -\theta_{i}\right)}{\sigma_{\rho i}^{2}}$$

Then, substitute the initial value $\hat{D}$ into Equation (16), and the cost function becomes a nonlinear function. Damping Guass–Newton method is adopted to solve the following equations,

(18) $$G_{T}\left(\hat{\Phi }+\partial \Phi \right)=\sum_{i=1}^{N}\frac{{\left(r_{i}\cos\left(\hat{\Phi }+\partial \Phi -\theta_{1}\right)-\hat{D}\right)}^{2}}{\sigma_{r}^{2}\cos^{2}\left(\hat{\Phi }+\partial \Phi -\theta_{i}\right)}$$

(19) $$\partial \Phi =-\frac{G_{T}^{\prime }}{G_{T}^{\prime \prime }}$$

Judge whether ∂Φ is less than the threshold value. If not, the result obtained through Equations (8) and (9) will be used as the initial value to repeat from Equation (17) to Equation (18) until it converges finally.

### 2.3. P3P Problem

In Section 2.2, we get the parameters of the three intersecting lines $l^{k}\left(k=1,2,3\right)$. Suppose that two lines among the three lines are $L^{i}$ and $L^{j}$ , and their parameters are denoted as $\left(D_{i},\Phi_{i}\right)$ and $\left(D_{j},\Phi_{j}\right)$. Because three planes of the trihedron are orthogonal with each other, three intersecting points denoted as $p_{k}\left(k=1,2,3\right)$ can be detected. The calculating equation can be represented as

(20) $$p_{k}={\left[\begin{array}{cc} \cos\Phi_{i} & \sin\Phi_{i}\\ \sin\Phi_{j} & \sin\Phi_{j} \end{array}\right]}^{-1}\left[\begin{array}{c} D_{i}\\ D_{j} \end{array}\right]\left(i,j,k=1,2,3,i<j\right)$$

Therefore, the distance between two of the three intersecting points can be obtained in the LRF coordinate system, which can be expressed as

(21) $$\left\{\begin{array}{c} d_{12}=∥p_{1}-p_{2}∥\\ d_{23}=∥p_{2}-p_{3}∥\\ d_{31}=∥p_{3}-p_{1}∥ \end{array}\right.$$

As described in Section 2.1, the three intersecting points of the laser line must be on the three edges or its extension lines of the trihedron. This means that the three intersecting points are on the three coordinate axes of the world coordinate system, so the three-dimensional coordinates of the three intersecting points in the world coordinate system can be expressed as

(22) $$Q_{k}=\lambda_{k}e_{k}\left(k=1,2,3\right)$$

where $e_{k}$ are the direction vectors of three coordinate axes, and $\lambda_{k}$ are the distances between the origin and the intersecting points. Now, we know the direction vectors of the three edges $e_{k}$, and the distances between two vertices of the trihedron are the same as $d_{12}$, $d_{13}$ and $d_{23}$. The unknown parameters are the distances between the origin and the intersecting points, i.e., $\lambda_{k},k=1,2,3$. Besides, three edges of the trihedron are perpendicular to each other. All the conditions above constitute a special P3P problem. The solution to this problem has been thoroughly studied in the literature [35], which is directly given here

(23) $$\left[\begin{array}{c} \lambda_{1}^{2}\\ \lambda_{2}^{2}\\ \lambda_{3}^{2} \end{array}\right]={\left[\begin{array}{ccc} 0 & 1 & 1\\ 1 & 0 & 1\\ 1 & 1 & 0 \end{array}\right]}^{-1}\left[\begin{array}{c} d_{23}^{2}\\ d_{13}^{2}\\ d_{12}^{2} \end{array}\right]$$

Equation (23) can only solve the absolute value of three edges $\lambda_{k}\left(k=1,2,3\right)$. Therefore, there are altogether eight possible solutions for the Equation (23) in mathematics. However, we can judge the sign from the actual location of the LRF and the trihedron, therefore obtaining the unique solutions of three edges $\lambda_{k}\left(k=1,2,3\right)$. It should be noticed that $\lambda_{k}\left(k=1,2,3\right)$ calculated by the above equation are not accurate when the angle formed by three planes is not a vertical angle.

In Section 2.1, we assumed that the laser scanning plane is the x−o−y plane of the LRF coordinate system. Therefore, the three-dimensional coordinates of the three intersecting points $P_{k}$ in the LRF coordinate system can be expressed as $P_{k}={\left[p_{k},0\right]}^{T}$. Because $P_{k}$ and $Q_{k}$ are the corresponding points in the LRF coordinate system and the world coordinate system, respectively, they satisfy the following relation,

(24) $$\begin{array}{cc} Q_{k} & =RP_{k}+t=\left[\begin{array}{cccc} r_{1} & r_{2} & r_{3} & t \end{array}\right]\left[\begin{array}{c} p_{k}\\ 0\\ 1 \end{array}\right]\\ \; & =\left[\begin{array}{ccc} r_{1} & r_{2} & t \end{array}\right]\left[\begin{array}{c} p_{k}\\ 1 \end{array}\right]\left(k=1,2,3\right) \end{array}$$

In Equation (24), R and t represent the rotation matrix and translation vector from the LRF coordinate system to the world coordinate system, respectively, and $r_{1}$, $r_{2}$, and $r_{3}$ represent the first, second, and third columns of the rotation matrix, respectively. In the Equation (24), R and t can be directly solved by matrix operation. The result is expressed as

(25) $$\left[\begin{array}{ccc} r_{1} & r_{2} & t \end{array}\right]=\left[\begin{array}{ccc} Q_{1} & Q_{2} & Q_{3} \end{array}\right]{\left[\begin{array}{ccc} p_{1} & p_{2} & p_{3}\\ 1 & 1 & 1 \end{array}\right]}^{-1}$$

(26) $$R=\left[\begin{array}{ccc} r_{1} & r_{2} & r_{1}\times r_{2} \end{array}\right]$$

To sum up, we construct a special P3P problem through the three intersecting points that are generated by observing a trihedron. Then, by solving this P3P problem, we obtain the coordinates of three intersecting points in the LRF coordinate system and the world coordinate system, respectively. Last, we get the extrinsic parameters of the LRF coordinate system relative to the world coordinate system.

### 2.4. LRFs Calibration Procedure

**Figure 4:**
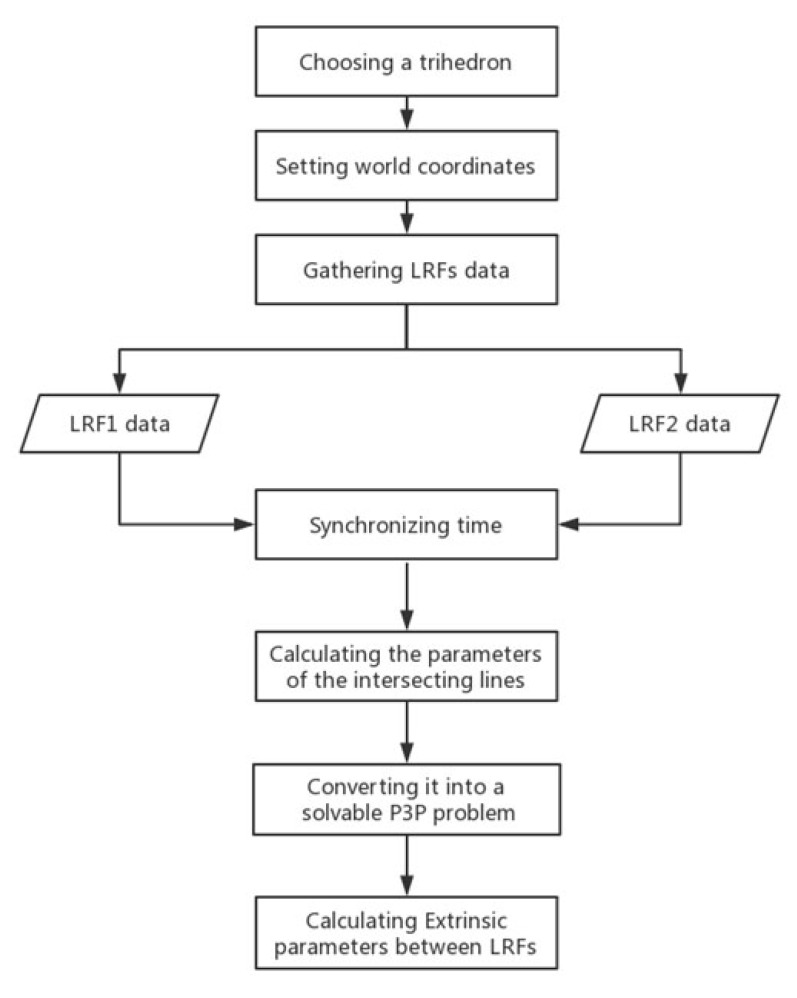
The process of calibration.

Section 2.2 and Section 2.3 specifically describe the method of determining the extrinsic parameters between the world coordinate system and the LRF coordinate system. As for two LRFs, we first get extrinsic parameters between the two LRFs and the trihedral world coordinate system using this method, and then we calculate the extrinsic parameters between two LRFs according to the method described in Section 2.1. The overall processes are shown in Figure 4, which mainly includes three important steps: laser data collection, laser points fitting, and extrinsic calibration.

Step 1: Laser data collection. A trihedron, such as a cube corner or a cubic prism, must be found in an indoor or outdoor scene. Then, establish a proper world coordinate system according to the edges of the trihedron. After that, adjust the position of the multiple LRFs devices or the scanning angle of the multiple LRFs to satisfy the condition so that these LRFs can observe the three planes of the trihedron.

Step 2: Laser points fitting. As the laser data collected in the first step are not accurate due to the laser ranging noises, we introduce a method called WI, which takes the variance of the distance from laser points to the actual line as the weight and iteratively calculate the parameters of the intersecting lines on three planes.

Step 3: Extrinsic calibration. After the parameters of the lines on three planes are determined, the three intersecting points between them can be directly calculated. Then, they are converted into a solvable P3P problem according to the method in Section 2.3, and the extrinsic parameters between the LRF coordinate system and the world coordinate system are calculated. All LRFs’ extrinsic parameters in the world coordinate system can be determined using the same method, and the extrinsic parameters between LRFs can be calculated by Equation (2).

## 3. Experiments and Discussion

To verify the accuracy and robustness of our method in sophisticated environments, we have carried out a large number of experiments in the simulation environment and the real environment. These experiments show that our method can effectively reduce the influence of the ranging noise of LRFs and achieve more accurate and robust extrinsic parameters than other methods.

Notably, although the corner trihedron is common and easy to find, it could be imperfect due to the errors of how well the three planes are perpendicular to each other (even the temperature may influence its shape), and finally influences the calibration accuracy. However, Hu et al. [36] performed statistical experiments on the impact of the angle between the three planes. Fan et al. [37] not only analyzed the impact of this deviation from orthogonality in their Figure 5, but also demonstrated that the small deviation has little impact on the calibration accuracy in their real experiments. Therefore, imperfect trihedron in practice is not discussed in this paper for both the simulation and real experiments.

### 3.1. Simulation Experiment

**Figure 5:**
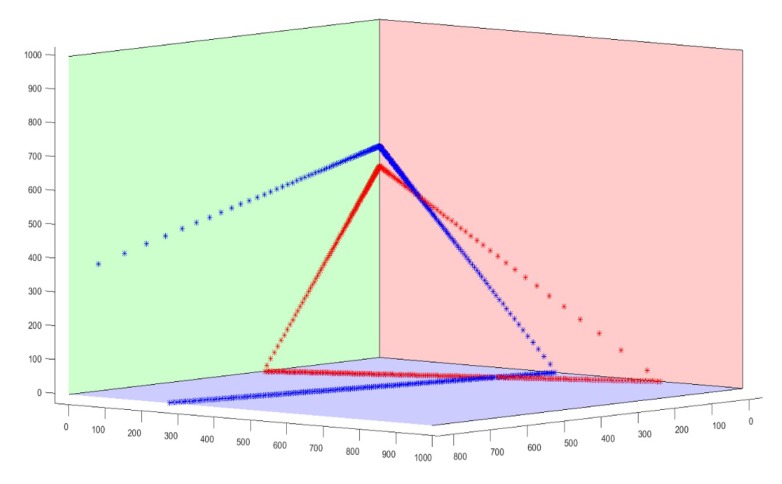
Schematic diagram of emulational laser points.

In the simulation experiment, we simulated the extrinsic parameters calibration between two LRFs, called LRF1 and LRF2. First, three 1 m × 1 m planes that are perpendicular to each other constituted a trihedron. The intersecting lines of the three planes were used to establish the world coordinate system. The LRF scanning parameters were set according to the actual LRF devices (Hokuyo UTM-30lx-em), which were used in the real data experiment. The scanning angle of the LRFs was 270 degrees and the scanning Angle resolution was 0.25 degrees. Second, we set the position and posture of two LRFs in the world coordinate system. Among them, the rotation angle vector between the LRF1 coordinate system and the world coordinate system was set as $\left[\begin{array}{ccc} -1.0 & -0.2 & 2.5 \end{array}\right]$ degrees, and the translation vector between them was $\left[\begin{array}{ccc} -30 & -110 & 700 \end{array}\right]$ mm. The rotation angle vector between the LRF2 coordinate system and the world coordinate system was set as $\left[\begin{array}{ccc} -0.2 & -0.1 & 2.5 \end{array}\right]$ degrees, and the translation vector between them was $\left[\begin{array}{ccc} -110 & -30 & 700 \end{array}\right]$ mm. Third, we generated the simulation data. We simulated the working process of the two LRFs and obtained the laser points, which were the intersection points between the laser beams and the trihedral planes. Then, adding zero-mean-value random noises with Gaussian distribution into the laser points data. To verify the performance of our method under different noise level, the distance values of simulated LRFs were added Gauss noise with a mean value of zero and a standard deviation from 3 mm to 30 mm. The simulation data of the two LRFs and a trihedron are shown in Figure 5, where the quadrilateral of three different colors represent three planes of the trihedron, the red star-shaped points represent the simulation data of LRF1, and the blue star-shaped points represent the simulation data of LRF2.

We mainly verify the performance of the proposed method in two aspects. The first purpose is to verify whether our method can fit the laser points with ranging error into a line accurately. The second purpose is to verify whether the extrinsic parameters between the two LRFs are accurate and robust.

#### 3.1.1. Evaluating the Parameters of Lines

In the simulation experiment, the parameters of the intersecting lines between the scanning plane and the trihedron can be uniquely determined after the LRFs’ position and posture are set. The ground truth of three lines are denoted as $\left(D_{k}^{true},\Phi_{k}^{true}\right)\left(k=1,2,3\right)$. To verify the accuracy of three lines, we fitted each laser points with the LS method, TLS method, and the WI method proposed in this paper, respectively. We set the mean of the absolute value of the angle and distance error of three-line parameters as evaluating indicators. That is,

(27) $$\begin{array}{cc} \varepsilon_{D} & =\frac{1}{3}\sum_{k=1}^{3}\left|D_{k}^{true}-D_{k}\right|\\ \varepsilon_{\Phi } & =\frac{1}{3}\sum_{k=1}^{3}\left|\Phi_{k}^{true}-\Phi_{k}\right| \end{array}$$

**Figure 6:**
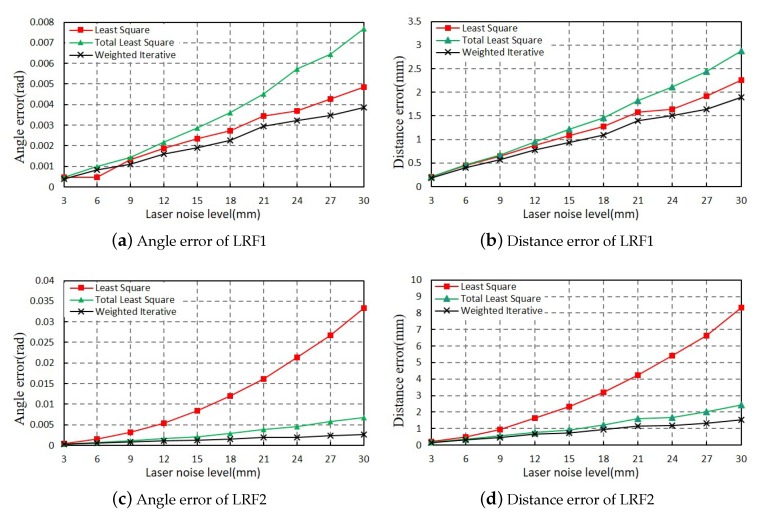
Linear fitting errors of two LRFs using three methods. Panels (**a**,**b**), respectively, represent the angle and distance error of the linear fitting parameters of LRF1, and panels (**c**,**d**), respectively, represent the angle error and distance error of the linear fitting parameters of LRF2.

The experiments were conducted under different Gaussian noise levels from 3 mm to 30 mm. At each noise level, the experiments were repeated for 100 times, and the mean value of the evaluating indicators was calculated. The results were drawn in Figure 6. Among them, the angle and distance errors of LRF1 are shown in Figure 6a,b and the angle and distance errors of LRF2 are shown in Figure 6c,d. The result of LS method, TLS method, and WI method are, respectively, represented by red square, green triangle, and black cross. It can be seen that with the continuous increase of ranging noise, the angle errors and distance errors of the fitting line are increasing in three methods.

As shown in Figure 6a,b, while the noise level of laser points increases from 3 mm to 30 mm, the average angle and distance errors of line fitting using three different methods increase in different speeds. Among them, the angle and distance errors of the TLS method increase in the fastest speed, followed by the LS method, and the WI method proposed in this paper has the slowest speed. When the noise level of laser points reaches to 30 mm, our method can achieve 0.004 angle error and 1.9 mm distance error, which are much smaller than the LS method and TLS method. The simulation experiments indicate that our method is more accurate than the other two methods, especially under high noise level.

The average angle and distance errors of LRF2 is different from LRF1 as the noise level of laser points increases from 3 mm to 30 mm. Compared with the other two methods, the errors of the LS method increase rapidly, and it achieves 0.034 angle error and 8.1 mm distance error when the noise level reaches to 30 mm. The reason for this phenomenon is that the LS method is not robust when the laser line is nearly parallel to the y-axis of the laser coordinate system. From the Figure 6c,d, the angle and distance errors of the LS method increase in the fastest speed, followed by the TLS method, and the WI fitting method proposed in this paper has the slowest speed. When the noise level of laser points reaches 30 mm, our method can achieve a 0.003 angle error and 1.8 mm distance error, which are approximately the same as the line errors of LRF1. Therefore, our method is more robust than the other two methods under a variety of situations.

#### 3.1.2. Evaluating Extrinsic Parameters

In Section 3.1.1, we have verified that the errors of resultant line parameters keep at a lower level, compared with the LS method and TLS method. In this section, we evaluated the accuracy of extrinsic parameters between two LRFs under different ranging noise levels. The extrinsic parameters between two LRFs gained by our method were denoted as R, t and the true value were denoted as $R^{\mathrm{true}},t^{\mathrm{true}}$. In the experiment, the difference in rotation matrix was measured by the Euler angle of error matrix, which is the product between the true rotation $R^{\mathrm{ture}}$ and the inverse of the measured rotation R. The difference in translation vector was measured by the norm between the true translation $t^{\mathrm{ture}}$ and the measured translation t. As is shown in Equation (28):

(28) $$\begin{array}{c} \varepsilon_{R}=∥R2E(R^{\mathrm{true}}R^{-1})∥\\ \varepsilon_{t}=∥t^{\mathrm{true}}-t∥ \end{array}$$

where $R^{\mathrm{true}}R^{-1}$ represents the error matrix between the true rotation $R^{\mathrm{true}}$ and the measured rotation R and the R2E() function transforms the rotation matrix into the form of Euler angle.

**Figure 7:**
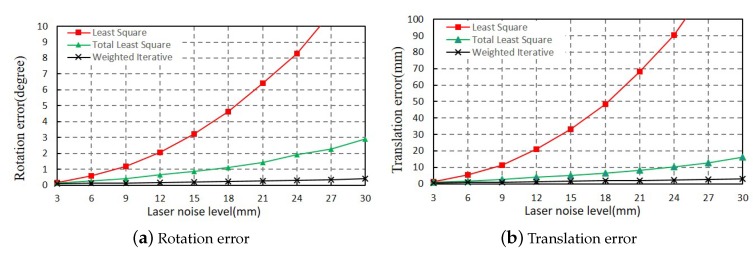
Calibration error of extrinsic parameters between two LRFs: (**a**) Rotation error and (**b**) translation error.

Similar to Section 3.1.1, we conducted the experiments at each ranging noise level from 3 mm to 30 mm. The above experiments were repeated 100 times, and the mean errors of the extrinsic parameters were calculated using the LS method, TLS method, and WI method. The results are shown in Figure 7, where Figure 7a,b, respectively, show the error of the rotation matrix and the translation vector. Generally, both the rotation and translation error of the proposed WI method are the least among the three methods, and they change little with the increase of ranging noise. As shown in Figure 7, when the ranging noise level is increased from 3 mm to 30 mm, the rotation error of the Euler angle increased from 0.07 degrees to 0.38 degrees, and the error of the translation vector increased from 0.59 mm to 2.95 mm. Compared with the ranging noises added on the two LRFs, the errors of the extrinsic parameters were always kept at a low level. Even if ranging noises with variance 30 mm were added to the data, the rotation and translation error of the proposed method were less than 0.4 degrees and 3 mm, respectively.

As there are no open source codes about the calibration of multiple LRFs, it is hard to do the same experiments by the methods introduced in literature review. We directly compare our rotation error and translation error with the result published in [17], where Choi et al. conducted similar simulation experiments. The result is shown in Table 1.

As shown in Table 1, we compare our method with the state-of-the-art method of Choi’s method [17] at different noise levels. The result shows that our method is much better than the initial value estimated by Choi’s method, and becomes very close to the optimized value. Moreover, it is robust to different noise levels. Additionally, the result of our simple method provides better initial parameters for the joint optimization, which will help a lot to optimize the extrinsic parameters further if a better accuracy is needed.

### 3.2. Real Data Experiment

Simulation experiments showed that our method had a good performance in calibrating extrinsic parameters between two LRFs under different ranging noise levels. We then verified the applicability of our method with the laser data collected in the real environment. The LRFs we used in the experiment were Hokuyo UTM-30lx-ems (Hokuyo, Tokyo, Japan), which were widely used in many SLAM applications. The scanning resolution of the LRF is 0.25 degrees, and 1080 measuring points can be gained in the 270 degrees scanning range every 25 ms. The maximum scanning range of the LRF is 60 m. In particular, the ranging noise of the laser points within the measurement distance from 0.1 to 10 m is ±30 mm, and that of the laser points within the measurement distance from 10 m to 30 m becomes ±50 mm. The specific parameters can be seen in Table 2.

**Figure 8:**
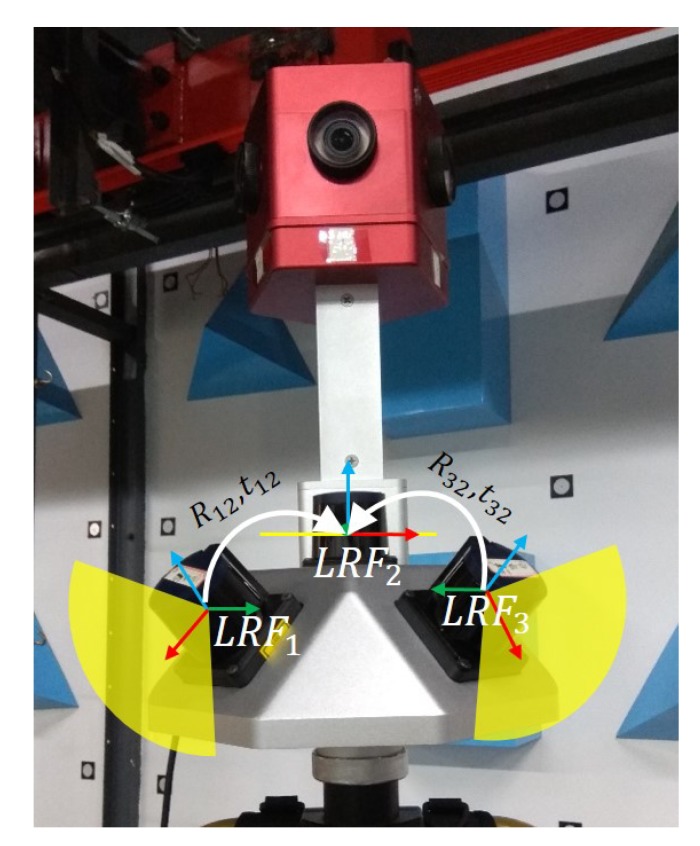
The SLAM device we used in the real experiment. Three LRFs on the SLAM device are denoted as LRF1, LRF2, and LRF3 from left to right, respectively. Assuming that the LRF2’s coordinate system is the reference coordinate system, the task of calibrating extrinsic parameters is to get the transformation relationships between the LRF1, LRF3 coordinate system, and the reference coordinate system.

As shown in Figure 8, three LRFs on SLAM devices are denoted as LRF1, LRF2, and LRF3 from left to right, respectively. Among them, the scanning plane of the LRF1 is oblique right, the scanning plane of the LRF2 is horizontal, and the scanning plane of LRF3 is oblique left. With the ranging center as the origin and the scanning plane as the x−o−y plane, each LRF coordinate system is established. Assuming that the LRF2’s coordinate system is the reference coordinate system, the task of extrinsic parameters calibration is to get the transformation relationships between the coordinate system of LRF1 and LRF3 and the reference coordinate system.

Based on the design specification for the SLAM integration, we got the initial values of extrinsic parameters through such 3D CAD (computer-aided design) software as Solidworks, which were carefully refined with the help of the camera above the LRF2. The refinement was conducted in a high-precision photogrammetric control field, and was demonstrated to achieve the state-of-the-art result of calibration in our previous work [37]. Denote the true values of extrinsic parameters as $T_{12}^{\mathrm{true}}\left[R_{12}^{\mathrm{true}}|t_{12}^{\mathrm{true}}\right]$ and $T_{32}^{\mathrm{true}}\left[R_{32}^{\mathrm{true}}|t_{32}^{\mathrm{true}}\right]$, respectively, we have

(29) $$\begin{array}{cc} R_{12}^{\mathrm{true}} & =\left[\begin{array}{ccc} 0.710 & -0.488 & 0.508\\ -0.006 & 0.716 & 0.697\\ -0.704 & -0.498 & 0.505 \end{array}\right]t_{12}^{\mathrm{true}}=\left[\begin{array}{c} 68.500\\ 44.330\\ 92.570 \end{array}\right]\left(mm\right)\\ R_{32}^{\mathrm{true}} & =\left[\begin{array}{ccc} 0.722 & 0.488 & -0.495\\ 0.006 & 0.706 & 0.707\\ 0.693 & -0.512 & 0.505 \end{array}\right]t_{32}^{\mathrm{true}}=\left[\begin{array}{c} -68.500\\ 44.330\\ 92.570 \end{array}\right]\left(mm\right) \end{array}$$

**Figure 9:**
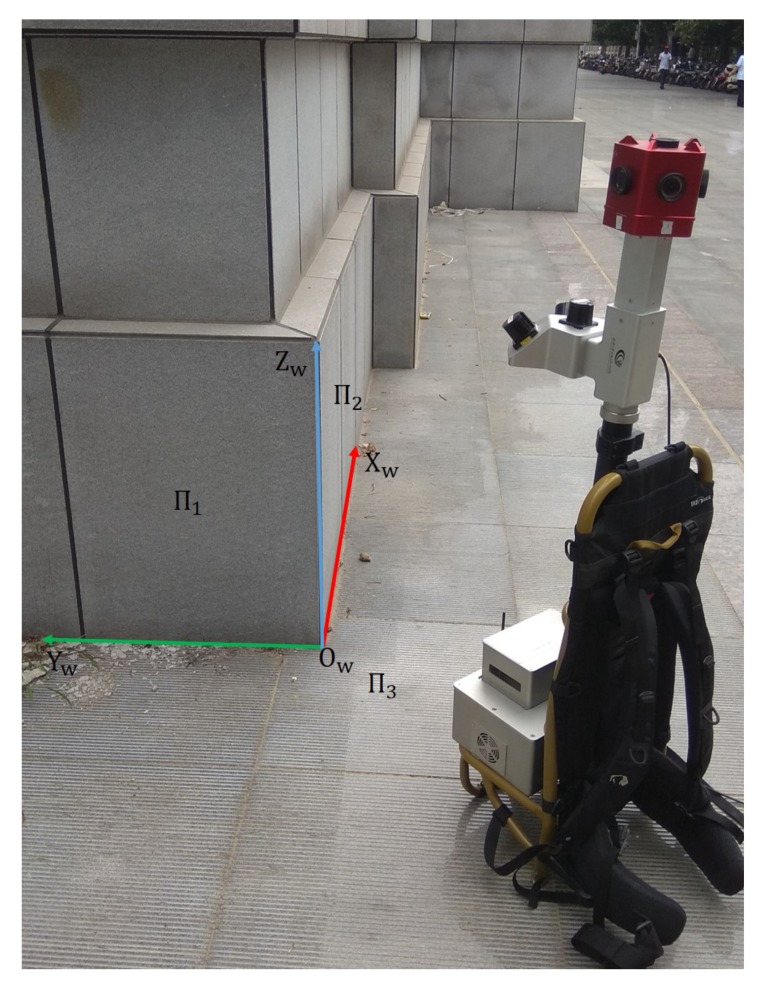
Real data experiment at the outdoor scene. Two planes of the building and the ground together constitute the trihedron described in Section 2.1. The three planes are denoted as $\Pi_{1}$, $\Pi_{2}$, and $\Pi_{3}$, and the world coordinate system $O_{W}-X_{W}Y_{W}Z_{W}$ is established by taking three edges of the trihedron as *X*, *Y*, and *Z* axis and the vertex of the trihedron as the origin.

In the following experiments, we selected a common vertical corner as the calibration pattern at an outdoor building. As shown in Figure 9, two planes of the building and the ground together constitute the trihedron described in Section 2.1. Three planes are denoted as $\Pi_{1}$, $\Pi_{2}$, and $\Pi_{3}$, and the world coordinate system $O_{W}-X_{W}Y_{W}Z_{W}$ is established by taking three edges of the trihedron as *X*, *Y*, and *Z* axis and the vertex of the trihedron as the origin.

**Figure 10:**
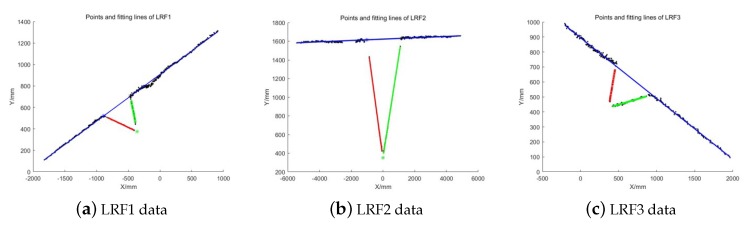
Results of WI fitting, the laser data, and fitting lines of (**a**) LRF1, (**b**) LRF2, and (**c**) LRF3, shown from left to right.

A group of laser data was collected with three LRFs of the SLAM device, and the linear equations of laser points on three planes were fitted by the proposed WI method. The result is shown in Figure 10, the laser data and fitting lines of LRF1, LRF2, and LRF3 were shown from left to right. The black points represent the laser points on the three planes of the trihedron, and the red, green, and blue lines represent the line on the plane $\Pi_{1}$, $\Pi_{2}$, and $\Pi_{3}$, respectively.

Then, according to the steps in Section 2.4, we calculated the extrinsic parameters between LRF1, LRF2, and LRF3. Let the extrinsic parameters calculated by the proposed WI method be denoted as $R_{12}^{\mathrm{WI}}$,$t_{12}^{\mathrm{WI}}$, $R_{32}^{\mathrm{WI}}$, and $t_{32}^{\mathrm{WI}}$, we have

(30) $$\begin{array}{cc} R_{12}^{\mathrm{WI}} & =\left[\begin{array}{ccc} 0.712 & -0.486 & 0.506\\ -0.013 & 0.711 & 0.702\\ -0.702 & -0.507 & 0.499 \end{array}\right]t_{12}^{\mathrm{WI}}=\left[\begin{array}{c} 60.859\\ 20.460\\ 83.583 \end{array}\right]\left(mm\right)\\ R_{32}^{\mathrm{WI}} & =\left[\begin{array}{ccc} 0.723 & 0.487 & -0.488\\ -0.001 & 0.708 & 0.705\\ 0.689 & -0.509 & 0.513 \end{array}\right]t_{32}^{\mathrm{WI}}=\left[\begin{array}{c} -65.042\\ 28.101\\ 72.882 \end{array}\right]\left(mm\right) \end{array}$$

**Figure 11:**
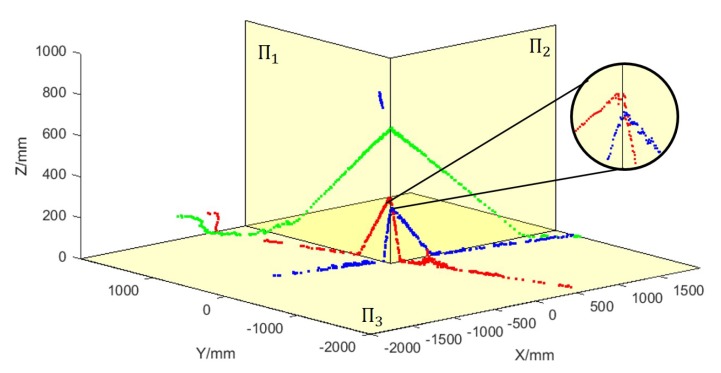
Results of three LRFs’ points inverted into the world coordinate system.

To visualize the calibration results, we converted all the laser points in their own LRF coordinate system to the world coordinate system and displayed them together in a figure. As is shown at Figure 11, three yellow planes respectively represent the three planes $\Pi_{1}$, $\Pi_{2}$, and $\Pi_{3}$ of the actual trihedron. The red points represent the laser data collected by LRF1, the green points represent the data collected by LRF2, the blue points represent the data collected by LRF3, and the figure in the black circle is the enlarged image of an area. From the image, it can be found that the laser points collected by the three LRFs coincides with the planes of the actual trihedron, which indicates that the extrinsic parameters of the three LRFs accurately restore their actual positions and orientations in the world coordinate system.

To further demonstrate the accuracy of the proposed WI method, extrinsic parameters calculated by the LS and TLS method were also given. Denote the extrinsic parameters calculated by LS method as $R_{12}^{\mathrm{LS}}$, $t_{12}^{\mathrm{LS}}$, $R_{32}^{\mathrm{LS}}$ and $t_{32}^{\mathrm{LS}}$, and those by TLS method as $R_{12}^{\mathrm{TLS}}$, $t_{12}^{\mathrm{TLS}}$, $R_{32}^{\mathrm{TLS}}$ and $t_{32}^{\mathrm{TLS}}$, respectively, we have

(31) $$\begin{array}{cc} R_{12}^{\mathrm{LS}} & =\left[\begin{array}{ccc} 0.711 & -0.487 & 0.505\\ -0.020 & 0.705 & 0.709\\ -0.702 & -0.515 & 0.491 \end{array}\right]t_{12}^{\mathrm{LS}}=\left[\begin{array}{c} 60.610\\ 7.599\\ 81.101 \end{array}\right]\left(mm\right)\\ R_{32}^{\mathrm{LS}} & =\left[\begin{array}{ccc} 0.724 & 0.487 & -0.487\\ -0.005 & 0.703 & 0.711\\ 0.689 & -0.517 & 0.506 \end{array}\right]t_{32}^{\mathrm{LS}}=\left[\begin{array}{c} -65.696\\ 23.815\\ 69.454 \end{array}\right]\left(mm\right) \end{array}$$

(32) $$\begin{array}{cc} R_{12}^{\mathrm{TLS}} & =\left[\begin{array}{ccc} 0.709 & -0.493 & 0.502\\ -0.021 & 0.698 & 0.715\\ -0.703 & -0.518 & 0.485 \end{array}\right]t_{12}^{\mathrm{TLS}}=\left[\begin{array}{c} 60.694\\ 3.228\\ 82.414 \end{array}\right]\left(mm\right)\\ R_{32}^{\mathrm{TLS}} & =\left[\begin{array}{ccc} 0.722 & 0.493 & -0.483\\ 0.006 & 0.694 & 0.719\\ 0.691 & -0.522 & 0.498 \end{array}\right]t_{32}^{\mathrm{TLS}}=\left[\begin{array}{c} -66.620\\ 8.510\\ 71.397 \end{array}\right]\left(mm\right) \end{array}$$

Based on the true values of extrinsic parameters, we calculated rotation and translation errors using the same measure of Equation (28) as simulation. The quantitative results are shown in Table 3. Bold text highlight the smallest result.

The table shows the result of calculating the extrinsic parameters error between LRF1, LRF2, and LRF3. The rotation error between LRF1 and LRF2 is denoted as $\varepsilon_{R_{12}}$, and the value calculated by our method is 0.610°, which is smaller than the rotation errors 1.231° and 1.685° calculated by the LS and TLS method, respectively. Similarly, the translation errors between LRF1 and LRF2 are denoted as $\varepsilon_{t_{12}}$, and our method can achieved 26.625 mm error which is smaller than 39.279 mm and 43.050 mm calculated by LS and TLS method. The rotation errors between LRF3 and LRF2 are denoted as $\varepsilon_{R_{32}}$, which are 0.523°, 1.019°, and 0.643° calculated by LS, TLS, and our method. The translation errors between LRF3 and LRF2 are denoted as $\varepsilon_{t_{32}}$, which are 31.033 mm , 41.652 mm, and 25.747 mm calculated by LS, TLS, and our method, respectively. The error calculated by LS method is the smallest, but the translation error is bigger than the value calculated by our method. The result proved that our method can get the extrinsic parameters between two LRFs with rotation error less than 1° and translation error less than 30 mm, with no need for an extra sensor. It is accurate and simple to calibrate the extrinsic parameters between two or more LRFs.

## 4. Conclusions and Future Work

This paper proposed a flexible, high-precision, and robust calibration method for multiple LRFs. This method uses a trihedron that is common in both indoor and outdoor scenes as a pattern. Without the need for other sensors, all that is required for our method is to find a position where all the LRFs can observe the trihedron. We made two contributions in this paper: First, we solved the problem of fitting lines with noisy laser points. Based on the assumption that the ranging noise of the laser points is regarded as a random variable with Gaussian distribution, our WI method was derived. By conducting a large number of simulation experiments, we proved that the linear parameters obtained by our method can achieve higher accuracy than traditional methods like LS and TLS. Even in the case that the ranging noise is very high, our method can obtain a relatively reliable calibration result. The second contribution is that we proposed a flexible and simple method to solve the extrinsic parameters between two LRFs.

**Figure 12:**
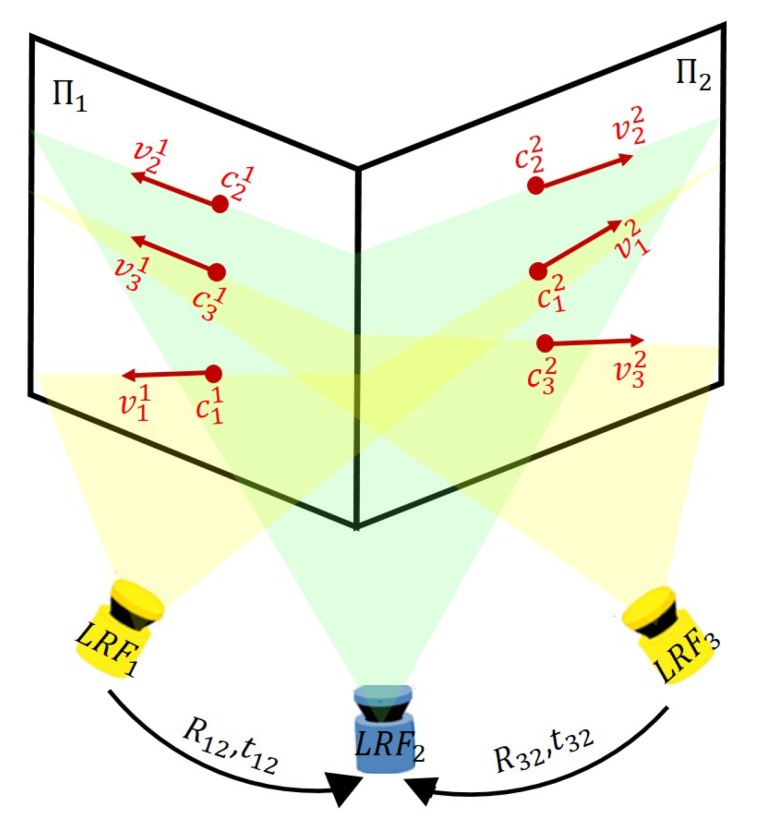
The coplanarity and orthogonality constraints established from a V-shaped wall.

To take full advantage of our simple method, the future work is mainly focused on improving the accuracy of calibration by jointly optimizing multiple LRFs with many redundant observations. As shown in our experiments, our method can get an accurate value of the extrinsic parameters of multiple LRFs in a simple way. When we need a better accuracy, the value calculated by our method can be used as an initial value for the joint optimized methods. A plane or sphere is enough to establish the constraints of optimization between LRFs. Inspired by the work in [17], a V-shaped wall with two intersecting planes, as shown in Figure 12, is also suggested to establish such constraints as coplanarity and orthogonality, where the scanning planes of LRF1, LRF2, and LRF3 can intersect both planes of a V-shaped wall, and the intersecting line $l_{i}^{j}$ between the LRFi’s scanning plane and the plane $\prod_{j}$ of the V-shaped wall can be represented by a center point $c_{i}^{j}$ and a direction vector $v_{i}^{j}$ fitted by our WI method.

## Figures and Tables

**Table 1:** The rotation error and translation error of the LRFs’ extrinsic parameters calibrated by our method and Choi’s method.

LRF’s Noise	Initial Value of Our Method		Initial Value of Choi’s Method		Optimized Value of Choi’s Method	
	$\varepsilon_{R}$ (°)	$\varepsilon_{t}$ (mm)	$\varepsilon_{R}$(°)	$\varepsilon_{t}$ (mm)	$\varepsilon_{R}$(°)	$\varepsilon_{t}$ (mm)
3 mm	0.07	0.59	0.25	16.10	0.01	0.41
6 mm	0.11	0.88	0.35	26.10	0.02	0.85
9 mm	0.13	1.08	0.40	31.50	0.03	1.10

**Table 2:** The technology performance of LRFs used in our experiment.

Item	Technology Performance
Measurement distance	0.1 to 30 m (White Kent Sheet) Maximum 60 m, 270°
Accuracy	0.1 to 10 m: ±30 mm 10 to 30 m: ± 50 mm
Scanning resolution	0.25° (360°/1440 steps)
Scanning time	25 msec/scan
Noise	<25 dB
Size	124 mm × 126 mm × 150 mm
Weight	About 210 g

**Table 3:** The rotation error and translation error of the LRFs’ extrinsic parameters calibrated by LS, TLS, and WI.

Method	$\varepsilon_{R_{12}}$ (°)	$\varepsilon_{t_{12}}$ (mm)	$\varepsilon_{R_{32}}$ (°)	$\varepsilon_{t_{32}}$ (mm)
LS	1.231	39.279	**0.523**	31.033
TLS	1.685	43.050	1.019	41.652
WI(our method)	**0.610**	**26.625**	0.643	**25.747**
